# Validating the psychiatric nurses methods of coping questionnaire: Arabic version

**DOI:** 10.1186/s12888-017-1573-y

**Published:** 2017-12-28

**Authors:** Ahmad Yahya AL-Sagarat, Marwa Barmawi, Lourance A. E. Al Hadid, Jamal A. S. Qaddumi, Lorna Moxham

**Affiliations:** 1 Community and Mental Health Nursing Department, Faculty of Nursing, Mutah University, Al-Karak, Jordan; 2 Faculty of Nursing- Al-Zaytoonah University of Jordan (ZUJ), Amman, Jordan; 3 Princess Aisha Bint Al-Hussein College of Nursing and Health Sciences, Al-Hussein Bin Talal University, Ma’an, Jordan; 4 Faculty of Medicine and Health Sciences, An-Najah National University, PO box 7, Nablus, Palestine; 5 School of Nursing, Faculty of Science, Medicine and Health, University of Wollongong, Northfields Ave, Wollongong, NSW 2522 Australia

**Keywords:** Coping skills, Psychiatric health nursing, Validity, Jordan, Pnmcq

## Abstract

**Background:**

The aim of the study was to undertake a psychometric analysis of the Psychiatric Nurses Methods of Coping Questionnaire (PNMCQ) - Arabic version when used to measure coping skills in psychiatric nurses in Jordan.

**Method:**

A descriptive, cross-sectional design was adopted in this study. A demographic questionnaire and the 35-item PNMCQ -Arabic were the measures used to collect data.

**Result:**

The PNMCQ demonstrated valid and reliable values when administered to psychiatric nurses in Jordan after it had been submitted to factor analysis.

**Conclusion:**

The development of PNMCQ: Arabic Version adequately measures coping skills in psychiatric nurses from a culturally appropriate context. Use of the tool can determine coping skills in psychiatric nurses with the view to positive staff development. Strategies identified based on results of the PNMCQ could ultimately result in better nurse retention and patient outcomes.

## Background

Recruiting appropriately educated nurses and then retaining them represents an ongoing challenge for the global healthcare industry [1]. Challenges related to working conditions and thus difficulty with staff retention could be a result of the considerable stress nurses encounter in their daily practice [2]. There are several factors that contribute to stressful working environments in the dynamically changing health care sector and include shift work, long working hours, a wide range and complex set of tasks and skills requiring critical decision making and autonomy. Further, the development and maintenance of therapeutic and collaborative interdisciplinary relationships with healthcare team members in an environment that is often resource poor, also contributes to stress [3]. Within nursing, different specialized areas have been noted as being particularly stressful. Critical care and psychiatric nursing are two examples of highly stressful discipline areas [4]. Of concern to psychiatric nurses is the growing burden related to administrative and organizational issues, increasing amounts of documentation particularly related to risk assessment, lack of consultation over work-related changes [5], and inadequate staffing. These issues are thought to take people away from the bedside (the reason people came in to the profession in the first place) and cause stress. In addition, work related stress levels are also exacerbated when nurses encounter escalating patient care needs [3], potentially violent patients and/or those who present with high levels of self-harming behavior [6, 7], role conflict, and when there is a perceived lack of supportive (social or professional) networks [4].

Psychiatric nurses are known to work in environments which contribute to additional sources of stress; the least of which is high patient-acuity levels [8]. Patients requiring treatment and care within unique environments like those in which psychiatric nurses work, are often involuntary in nature and as a consequence of their severe mental illness and high levels of distress, can be extremely challenging to manage [4]. Thus, for psychiatric nurses who work within the context of high stress [9], the potential to leave the profession, require counseling, and experience higher rates of sick leave, is exacerbated.

### Stress, coping and mental health nursing

Stress has been defined as “any circumstance that places special physical and/or psychological demands on a person such that an unusual or out-of-the-ordinary response occurs” [10]. If levels of stress are not managed appropriately, the expected results could be low job satisfaction, poor work performance, high absenteeism and turnover [11].

Psychology, as a discipline area pays particular attention to the emotional aspects of stress that represent the most prevalent symptoms [11]. Psychological stress refers to the negative emotional and cognitive states that occur when individuals believe the demands placed on them exceed their ability to adapt to their situations [12].

Psychiatric nurses need to manage their stress and keep themselves well to provide the best care they can for their patients. It is therefore, important for them to nurture good physical and mental health; instigating methods of coping that strengthen their ability to cope effectively. This in turn, will reduce the levels of stress and burnout they experience [13].

Therefore, understanding how psychiatric nurses cope with job related stress is an important workplace strategy, not only for the nurses themselves but also for the organizations they work for and ultimately the patients who are recipients of their care.

Coping was defined by Folkman et al. [14] as any cognitive or behavioral effort to manage, reduce, or tolerate events that individuals perceive as potentially threatening to their well-being.

There have been several studies that have investigated coping skills among mental health nurses [4, 8, 13, 15]. However, based on a thorough review of the literature, the researchers were unable to locate any developed or validated tools in Arabic that could be used for measuring coping skills among psychiatric nurses. Therefore, this study aimed to address the lack of validated symptom measures to assess the issue in Arabic countries by translating the Psychiatric Nurses Methods of Coping Questionnaire (PNMCQ) into Arabic. The focus of this research is on nurses who work in Jordan.

### The psychiatric nurses methods of coping questionnaire

The PNMCQ was developed specifically for use with psychiatric nurses working on inpatient units and was designed to measure coping skills [15]. Permission to use this tool was obtained by the researchers from the developer of the scale. The PNMCQ consists of 35 items comprising five subscales: diverting attention (9 items), self-regulation and self-attitude (6 items), social support at work (6 items), positive attitude toward work role (9 items), and emotional comfort (5 items). The PNMCQ is scored on a 5-point Likert scale: 1 = never; 2 = rarely; 3 = occasionally; 4 = often; and 5 = all the time [16]. As per the author of the scale, both face and content validity were established when the PNMCQ was development [16].

The questionnaire development commenced by collecting items from various sources, including interviews with nurses working in psychiatric units and the review of scales measuring coping strategies. In addition, some items in the PNMCQ were adopted from responses to a previous study by Carson et al. conducted on psychiatric nurses [17], who asked 568 psychiatric nurses the following question: what is the main method you use to help you cope with stress? Responses to this question yielded 108 items, which were then tested by another group of psychiatric nurses to refine the list exclusively to coping strategies; other items found irrelevant or insignificant during the second study were eliminated. There were 50 items which remained from the second study. These items were also tested on 76 nurses and the number was reduced to 35 items using psychometric analysis [15]. The PNMCQ was also tested for reliability and stability over time and use among psychiatric nurses in many studies. For instance, McElfatrick et al. [15] used the PNMCQ on 175 psychiatric nurses in Northern Ireland, and reported that the reliability value of the PNMCQ was 0.90, and alpha values for the subscales ranged from 0.67 to 0.78 [15].

## Methods

### Design and sample

A descriptive, cross-section design was used on a non-probability convenience sample. The participants were psychiatric nurses working in three public hospitals in Jordan. Ethical approval had been granted by the Jordanian Ministry of Health, Human Ethics Committee under the number (8526/13/8/2012). The study procedure and purpose were explained to the participants during visits by the research team to clinical settings, and each candidate was provided with a copy of the participant information sheet (PIS), which stated that participation was voluntary, and that participants could withdraw at any time without prejudice. The questionnaire did not contain any request for name or any other identifying information. The information sheet also included the researchers’ contact details to obtain any further information about the study from any of the researchers. Participants completed and returned the study questionnaire by dropping it in the hospital internal mail box. Data were collected during the period of June 2014 and December 2014.

### Translating the instrument

Three Arabic-speaking researchers with a PhD degree translated the source questionnaire. In collaboration with the translating researchers, the investigators clarified unclear items and revised items for cultural suitability. The agreed-upon revised version was sent to two Arabic speaking researchers for back-translation. The final version of the questionnaire was sent to a native English speaker to confirm the face validity of the back-translated items. The researchers then sent the Arabic version of the PNMCQ to a specialist in Arabic language to ensure readability and structuring of all items, and then Arabic version was ready for use.

### Statistical analysis

Data analyses were analyzed using IBM SPSS version 21. Sample characteristics and response on the PNMCQ were analyzed using measures of central tendency, including the means and standard deviation. The normal distribution of the questionnaire mean scores was tested using Kolmogorov-Smirnov goodness of fit statistic [18]. Exploratory factor analysis using an axial principal factoring with varimax rotation was adopted on the scale 35 items [19]^,^ which resulted in the extraction of seven factors. The cut-off point adopted in this study for item loading was 0.40 [20, 21].

### Reliability

Cronbach’s alpha for the 35-item scale was 0.948, demonstrating a high reliability value [16]. The Spearman-Brown coefficient was 0.939 for both equal and unequal length. The Guttman Split Half coefficient was 0.939, and Cronbach’s alpha for both parts were 0.899 and 0.900 respectively. Item correlation was 0.885, indicating an effective statistical item correlation.

## Results

The completed questionnaires were included for analysis only if all responses were completed. The number of completed questionnaires was 99 from a total of 150 distributed copies representing a response rate of 66%. The average age of nurses in this study was 34.8 years (range = 24–57). The number of male nurses was 58 (58.6%) and the females were 41 (41.4%). This represents a similar spread of gender employed as psychiatric nurses in Jordan. Participants’ qualifications included a diploma (35.4%) or a bachelor degree (59.6%) in Nursing. The majority (78.8%) of participants were married. Participants identified as working mainly in an acute (35.4%) or chronic (32.3%) mental health area. Most participants were registered nurses 76 (76.5%), and only 34 (34.3%) reported that they did not attend any workshop on mental health.

### Factor analysis

An exploratory factor analysis (EFA) was performed on the original 35 items. The first seven items reflected more than 66% of the total variance (8 values were equal to or more than 1). The Kaiser–Meyer–Olkin Measure of Sampling Adequacy was 0.878 indicating an acceptable level of inter-correlation among the items [22]. Bartlett’s test of sphercity showed that the correlations between the items were adequate to perform factor analysis, with an approximate Chi-Square of 986.059 (*p* < .001). Except for two items: ‘By knowing that I can depend on other members of staff’ and ‘By having the freedom to express my views openly’, the remaining inter-correlation among the 35 items was less than 0.30. Therefore, the level of inter-correlation among the items was acceptable as it did not affect the internal consistency [16]. High internal consistency values usually result from high inter-correlation among items on the scale indicating overlapping items.

## Discussion

Psychometric evaluation aims to measure reliability and validity of research scales by assessing the characteristics of scales [23]. The study aimed to translate the PNMCQ into Arabic, and the study aim was met. The scale demonstrated validity and reliability when administered to the study sample. When measuring the scale’s psychometric properties, both reliability and validity are considered the most essential characteristics [24]. The reliability of a scale refers to the degree to which this research instrument it yields similar or comparable results when used repeatedly [25]. In this study, the PNMCQ-Arabic achieved high reliability values which are comparable with results from previous studies. This indicates that results are consistent within itself and across time. Validity refers to truthfulness of the claim that the research scale measures what it says it actually measures [26]. This issue has been tested by the authors and confirmed by subsequent uses of the scale in different settings. Internal consistency of the PNMCQ-Arabic was tested using item analysis that examined the extent to which all the items of the scale measured the same factor [19]. The internal consistency of the scale was found to be reasonably high [23]. Internal consistency, however, does not provide adequate evidence on which to judge reliability; other measures exist confirming these findings, including test-retest measures.

By adopting suggestions made by Streiner and Norman [26], this study examined the construct validity of the PNMCQ using factor analysis, selecting items of powerful representation and setting a statistically acceptable cut-off value. These issues were carried out to ensure that the psychometric properties and construct validity of the scale were checked according to statistically acceptable measures. The deletion of items with weak representation, and irrelevant items substantially added to the statistically-supported values. A demographic questionnaire and the 35-item Arabic version of PNMCQ were the instruments used to collect the data.

The factor ‘Perceiving the role of self and the others’ were explained by ten statements.

Leary et al. [27] found that the psychiatric nurses utilized various methods for coping with stress. These methods included efficient time management, planned team meetings and support networks and improved communication channel both within and between professional disciplines and departments.

The factor ‘Self regulating and self control’ was explained in this study by six statements.

A study in Ireland [28] investigated stressors, burnout and coping strategies among 69 psychiatric nurses working in both hospital and community-based settings in Dublin. The participants completed the Mental Health Professional Stress Scale, the Maslach Burnout Inventory and the Psych Nurse Methods of Coping Scale. The researchers reported that the most frequently reported coping strategy undertaken by psychiatric nurses was diverting one’s attention’. McTierna and McDonald [28] indicated that avoidance strategy adopted by the nurses maintained their psychological well-being while providing their services as professionals.

The third factor, ‘Positive attitudes towards your role at work’ is explained by five statements. Researchers agree that the approach and attitude in which staff deal with consumers indirectly influences the ward atmosphere in the psychiatric ward [29]. A more detailed explanation may lie in the idea that an interactive relationship between the hospital staff and consumers allows the development of healthy interactions, which in turn results in a therapeutic environment inside the psychiatric ward [29]. Moreover, it is believed that “consumers are more likely to improve in programmes in which staff are more satisfied with their job and thus establish a more therapeutic environment” [29]. Yet another study [30] stated that optimistic people believe that things are generally going in the right track and they focus on positive aspects of events. Conversely, pessimistic people expect things to go bad and for there to be negative outcomes. Pessimists focus on negative aspects and harbor bad feelings. Optimists have a higher quality of life than pessimists.

‘Resources to emotional stability’ is the fourth factor and was explained by five statements in this study. In this vein, Dubrow-Marshall [31] highlighted the importance of nurses having healthy lifestyles separate from work. Moreover, Dubrow-Marshall [31] indicated that self-care sustains professionals’ own health and thus enables the provision of high-quality support [31].

The factor ‘Positive feeling about self and the surroundings’ was explained in this study by three statements. In this regard, White [32] conducted a correlational study to describe perceived occupational stressors, coping methods, and burnout levels of 46 psychiatric nurses working on locked psychiatric units in southeastern Michigan, USA. The study reported that the most frequently adopted coping strategies methods by psychiatric nurses included spending free time and practicing their favorite hobbies outside work, realizing that life outside of work is healthy, enjoyable and worthwhile, looking forward to going home at the end of the day, having a stable home life that is kept separate from their work life, and having confidence in one ability to do the job well.

The sixth factor is ‘Social support at work’ and was explained in this study by four statements. An examination of the literature [9] has identified three types of coping methods among hospital nurses. The first one was identified as a problem-solving method, which contains defining goals, planning and searching for alternative solutions. The second method was social support, which included the tendency to turn to others for advice, communication, and comfort. The final one, was the avoidance method, which involves physical or psychological withdrawal through distraction or fantasy [9].

Many sources of social support have been identified in the literature for health care professionals and nurses. Sources of social support came from work (i.e., supervisors, colleagues, and coworkers) and from home (i.e., family members, spouse, and friends) [33]. Amarneh, et al. [33] investigated the effect of social support from coworkers on job performance among Jordanian hospital nurses. The results showed that the perceived social support from coworkers enhanced the level job performance, decreases the level of job stress, and enhances work commitment. These findings were consistent with a more recent study, which indicated that social support buffers the impact of stress in psychiatric nurses, and thus encouraging personal accomplishment at work [34].

The final factor ‘Finding emotional comfort, was explained by two statements. In this regard, Akbar, Elahi, Mohammadi and Khoshknab [35] found that when nurses encountered challenges in their daily assigned duties and responsibilities for any given reason, in most cases they would approach a colleague, senior staff, or even a family member for help if they required informational support, indicating that informational and emotional comfort at work is an important strategy in coping with work-related stress in that culture.

## Conclusion

Coping strategies among psychiatric nurses has been widely researched internationally over many years. That said, most studies have been conducted in Western countries such as the United States of America and the United Kingdom. However, only limited studies on coping strategies among psychiatric nurses have been conducted in Arabic speaking countries, and specifically none in Jordan. Therefore, this study which aimed to undertake a psychometric analysis of the PNMCQ -Arabic version when used to measure coping strategies amongst mental health nurses in Jordan not only contributes results of its administration to the body of knowledge but the instruments translation and subsequent validation enables it to be used in other Arabic countries. The translated and validated instrument focused on measuring the coping strategies amongst nurses in psychiatric hospitals in Jordan. Given that work-related stressors are one of the main psychosocial risks for psychiatric nurses the development of a valid and reliable tool that can assess measuring the coping strategies is important. The validation of the PNMCQ-Arabic version can thus provide deep understanding from a culturally appropriate perspective which can lead to change. Such change could ultimately result in better patient outcomes.
